# Multi-country evaluation of the durability of pyrethroid plus piperonyl-butoxide insecticide-treated nets: study protocol

**DOI:** 10.1186/s12936-023-04465-x

**Published:** 2023-01-27

**Authors:** Emmanuel Mbuba, Olukayode G. Odufuwa, Jason Moore, Selemani Mmbaga, Emile Tchicaya, Constant Edi, Vani Chalageri, Sreehari Uragayala, Amit Sharma, Manju Rahi, Kamaraju Raghavendra, Alex Eapen, Hannah Koenker, Amanda Ross, Sarah J. Moore

**Affiliations:** 1 Vector Control Product Testing Unit, Environmental Health and Ecological Science, Ifakara Health Institute, P.O. Box 74, Bagamoyo, Tanzania; 2 Swiss Tropical and Public Health Institute, Kreuzstrasse 2, 4123 Allschwil, Switzerland; 3 University of Basel, St. Petersplatz 1, 4002 Basel, Switzerland; 4 Epidemiology and Population Health Department, London School of Hygiene & Tropical Medicine, Keppel Street, London, WC1E 7HT UK; 5 Swiss Centre for Scientific Research in Côte d’Ivoire, 1303 Abidjan, Côte d’Ivoire; 6 Vegro Aps, Copenhagen, Denmark Refshalevej 213A,; 7 Field Unit, ICMR-National Institute of Malaria Research, Bangalore, Karnataka India; 8 ICMR-National Institute of Malaria Research, Sector-8, Dwarka, New Delhi, 110077 India; 9 ICMR-Indian Council of Medical Research, Ansari Nagar, New Delhi, India; 10 Field Unit, ICMR-Indian Council of Medical Research, Chennai, India; 11 Tropical Health LLP, Baltimore, USA

**Keywords:** Insecticide-treated nets, Durability, Piperonyl-butoxide, Veeralin®, Tsara® Boost, Olyset® plus, MAGNet®

## Abstract

**Background:**

Mass distributions of long-lasting insecticidal nets (LLINs) have contributed to large reductions in the malaria burden. However, this success is in jeopardy due in part to the increasing pyrethroid-resistant mosquito population as well as low LLINs coverage in various areas because the lifespan of LLINs is often shorter than the interval between replenishment campaigns. New insecticide-treated nets (ITNs) containing pyrethroid and piperonyl-butoxide (PBO) have shown a greater reduction in the incidence of malaria than pyrethroid LLINs in areas with pyrethroid-resistant mosquitoes. However, the durability (attrition, bio-efficacy, physical integrity and chemical retainment) of pyrethroid-PBO ITNs under operational settings has not been fully characterized. This study will measure the durability of pyrethroid-PBO ITNs to assess whether they meet the World Health Organization (WHO) three years of operational performance criteria required to be categorized as “long-lasting”.

**Methods:**

A prospective household randomized controlled trial will be conducted simultaneously in Tanzania, India and Côte d’Ivoire to estimate the field durability of three pyrethroid-PBO ITNs (Veeralin®, Tsara® Boost, and Olyset® Plus) compared to a pyrethroid LLIN: MAGNet®. Durability monitoring will be conducted up to 36 months post-distribution and median survival in months will be calculated. The proportion of ITNs: (1) lost (attrition), (2) physical integrity, (3) resistance to damage score, (4) meeting WHO bio-efficacy (≥ 95% knockdown after 1 h or ≥ 80% mortality after 24 h for WHO cone bioassay, or ≥ 90% blood-feeding inhibition or ≥ 80% mortality after 24 h for WHO Tunnel tests) criteria against laboratory-reared resistant and susceptible mosquitoes, and insecticidal persistence over time will be estimated. The non-inferiority of Veeralin® and Tsara® Boost to the first-in-class, Olyset® Plus will additionally be assessed for mortality, and the equivalence of 20 times washed ITNs compared to field aged ITNs will be assessed for mortality and blood-feeding inhibition endpoints in the Ifakara Ambient Chamber Test, Tanzania.

**Conclusion:**

This will be the first large-scale prospective household randomized controlled trial of pyrethroid-PBO ITNs in three different countries in East Africa, West Africa and South Asia, simultaneously. The study will generate information on the replenishment intervals for PBO nets.

## Background

Long-lasting insecticidal nets (LLINs) have contributed substantially to the reduction of the malaria burden over the past 15 years [1]. Between 2004 and 2020, more than two billion LLINs have been distributed worldwide [2]. Since the 1980s [3, 4], bed nets have been treated with pyrethroid insecticide because it has low toxicity to humans and non-targeted arthropods, and has a rapid insecticidal effect against susceptible malaria mosquitoes [5, 6]. Pyrethroid LLINs provide a physical barrier between humans and host-seeking mosquitoes, and the pyrethroid kill, incapacitate (knockdown), or inhibit feeding among mosquitoes that come into contact with the net [7, 8], enhancing bite protection even when the net is physically damaged [9]. When used with high coverage, LLINs provide community protection against malaria by reducing mosquito survival [7, 10–12].

Pyrethroid-resistant malaria vectors are now a major threat to the effectiveness of LLINs [13]. To tackle this threat, a new generation of insecticide-treated nets (ITNs) have been developed using pyrethroids combined with a synergist, such as piperonyl-butoxide (PBO) [12, 14]. PBO has little insecticidal activity by itself but acts as a synergist by blocking the oxidases (cytochrome P450) that commonly detoxify pyrethroid insecticides inside the mosquito’s body to restore pyrethroid efficacy [15–18]. Pyrethroid-PBO ITNs were found to yield a greater reduction in the incidence of malaria cases compared to standard pyrethroid LLINs for up to 21 months of use in areas with a high level of pyrethroid-resistance in mosquitoes [19] and have shown increased mosquito mortality compared to pyrethroid only LLINs in experimental hut studies when unwashed or after 20 washes [20]. However, in the Cochrane review of pyrethroid-PBO ITNs, the authors concluded that there is no evidence that PBO content persists under operational conditions for three years [20].

Bed nets that retain their bio-efficacy thresholds (the proportion of mosquitoes (≥ 95%) knocked down after one hour or (≥ 80%) mortality after 24 hours for cone bioassay, or if cone bio-efficacy thresholds are unmet, the proportion of mosquitoes (≥ 90%) blood-feeding inhibition or (≥ 80%) mortality after 24 h for tunnel tests) for at least 20 World Health Organization (WHO) standard washes under laboratory conditions and three years of recommended use under operational conditions are qualified as LLINs [8, 14]. However, it is not known if the 20 times WHO laboratory washed ITNs correspond to the field-used ITNs in inducing mosquito mortality. Also, although the WHO has prequalified several pyrethroid-PBO ITNs, there is currently limited data available on their physical and insecticidal durability under operational conditions in different geographical regions [8]. Understanding the durability of pyrethroid-PBO ITNs under operational conditions is important to guide procurement decisions and for devising replacement policies [21].

The physical integrity of a LLIN under operational conditions is one of the key determinants of net retention in the household, and more than 75% of discarded LLINs have damage and are perceived as failing to protect the user [22]. Factors associated with the loss of the physical integrity of LLINs include the environment in which the net is used [23–25], the users’ net care behaviour and attitude towards the LLIN [26], the type of net material [27–30], snag strength, bursting strength, abrasion resistance and resistance to hole enlargement [31], the number and age of people sleeping under the net [32, 33], and the geographical location in which the net is used [34]. Therefore, it is recommended to conduct ITN durability studies in multiple geographical locations [8]. The current study aims to measure the insecticidal and physical durability of three PBO ITNs alongside a pyrethroid only net over three years of household use in three different regions.

## Study objectives

### Overall aim

To evaluate the physical and insecticidal durability of PBO ITNs (Veeralin®, Tsara® Boost, Olyset® Plus) and a standard pyrethroid LLIN (MAGNet®) over 3 years of household use in Tanzania, East Africa, Côte d’Ivoire, West Africa and India, South Asia using standard WHO methods [8], and additionally to assess the non-inferiority [35, 36] of the PBO ITNs against Olyset® Plus.

### Secondary objectives

i. Estimate the attrition rate of each ITN product at 6, 12, 24 and 36 months of household use in each location.
ii. Estimate fabric integrity of each ITN product at 6, 12, 24 and 36 months of household use in each location.
iii. Estimate the bio-efficacy of each ITN product after 6, 12, 18, 24, 30 and 36 months of household use in each location.
iv. Measure the insecticide content of each ITN product after 12, 24 and 36 months of household use in each location.
v. Assess non-inferiority of field used Veeralin® and Tsara® Boost compared to Olyset® Plus and superiority of PBO ITNs compared to pyrethroid only MAGNet® from the Tanzania site after 12 and 24 months, upon the 24-h mortality primary endpoint.
vi. Assess the equivalence of unwashed and 20 times laboratory washed nets and field-used ITNs after 12 and 24 months of each net type from the Tanzania site upon the 24-h mortality and blood-feeding inhibition endpoints.

## Methods

### Study area

This study will be conducted in selected villages in Tanzania, Côte d’Ivoire and India (Table 1). Study villages will be selected based on the availability of a large number of households required for the study, their accessibility throughout the year, the high abundance of malaria vectors that promotes mosquito net usage [37] and proximity to the bio-efficacy testing laboratory in each study country.

**Table 1:** Description of study location in Tanzania, Côte d’Ivoire and India

Features	Tanzania	Côte d’Ivoire	India
Location of the study	Bagamoyo district in the Coastal region, located 70 km North of Dar Es Salaam	Tiassalé district in the Agnéby-Tiassa region, located 135 km North of Abidjan, in Southern Côte d’Ivoire	Kurnool district in the Andhra Pradesh state. Study villages are on the embankment of the river Tungabhadra
Global positioning system	Latitudes 6° 37ʹ South Longitudes 38° 58ʹ East	Latitude 5° 54′ North Longitude 4° 50′ West	Latitude 15° 81′ North Longitude 77° 96′ East
Tropical Climate	An average annual rainfall of 900 mm and temperature of 28 °C	An average annual rainfall of 1739 mm and temperature of 26.6 °C	An average annual rainfall of 705 mm and temperatures ranging from 26 to 46 °C in the summer and 12 °C to 31 °C in the winter
Economic activities of inhabitants	Most of the inhabitants are small-scale farmers	Most of the inhabitants are subsistence farmers and civil servants	Most of the inhabitants are small-scale subsistence farmers and civil servants

### Study design

**Fig. 1:**
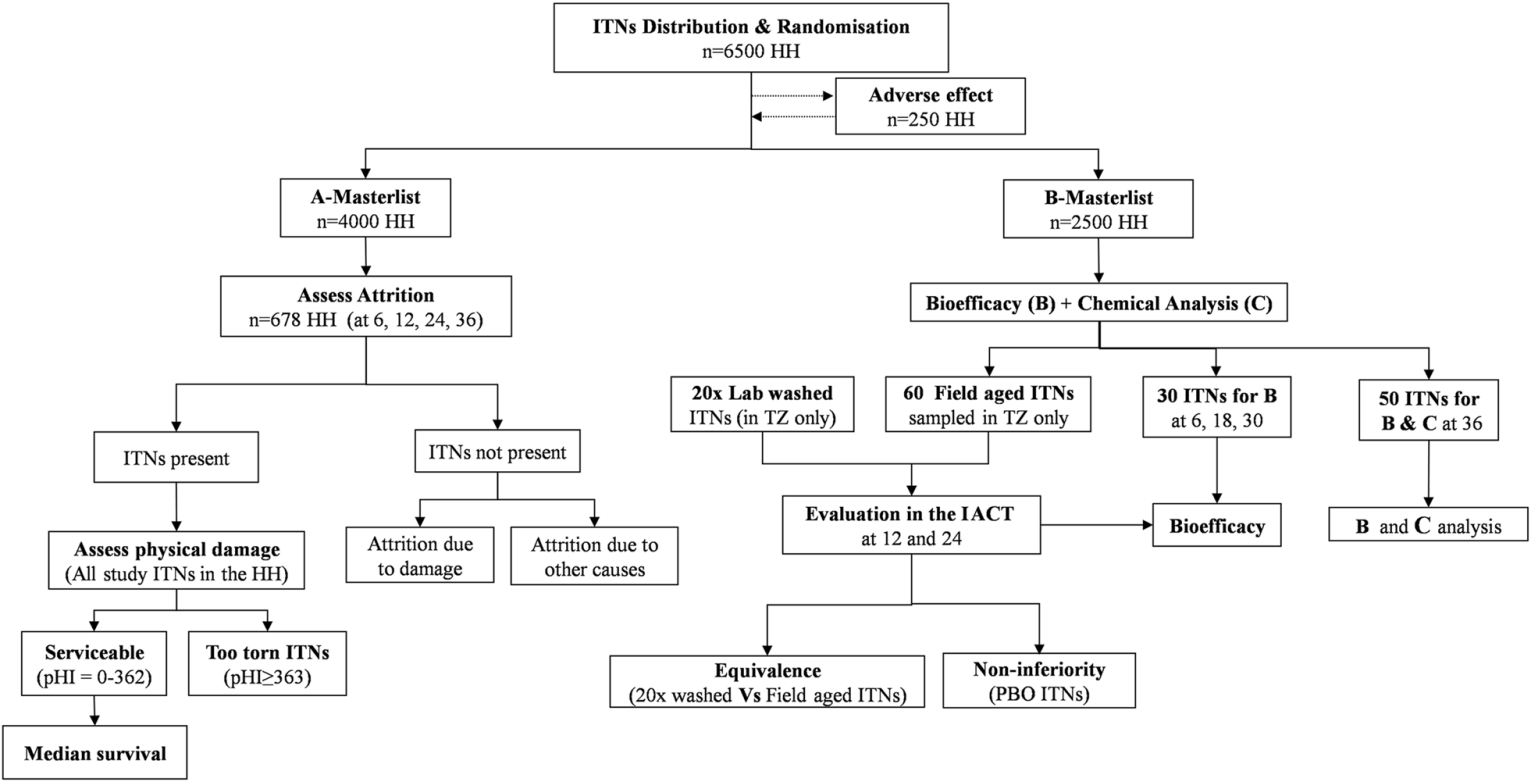
Study flow diagram showing the study procedures; attrition, fabric integrity assessment, bio-efficacy and chemical residual content will be done as per WHO guidelines [38]. The cross-sectional assessment of the non-inferiority and equivalences analysis will be conducted in Tanzania. *B* bioefficacy, *C* chemical analysis, *HH* household, *ITNs* insecticidal trated nets, *pHI* propotionate hole index, *TZ* Tanzania, *IACT* Ifakara Ambient Chember Test

This will be a prospective household-randomized controlled trial following WHO guidelines [8] for monitoring the durability of ITNs with slight modifications to the sample size and study procedures. A minimum of 6500 households from the selected villages in the study area will be enrolled in each country. In this study, a household is defined as a group of people who share living accommodation and who are eating from one pot. The households will be the unit of ITNs randomization and the individual nets as a unit of observation. At baseline, a household survey will be conducted to collect households’ information including demographic and socioeconomic characteristics and the location of the household. At the same time, mosquito nets will be distributed by a study team in each household to cover all sleeping places, to ensure maximum coverage of ITNs for all members of the household. Follow-up surveys will be conducted to assess the rate of net loss in the receiving households (attrition rates due to all causes, as well as for wear and tear), physical integrity, bio-efficacy and chemical residual content of ITNs collected from households at the time intervals up to 36 months (Table 2). The study flow diagram is shown below (Fig. 1). All participating households will be blinded to which net product they receive, and study investigators will also be blinded to intervention allocation.

**Table 2:** Schedule of activities

Activities	Month							
	0	1	6	12	18	24	30	36
Conducted at baseline								
Community sensitization	x							
Household recruitment	x							
Baseline questionnaire	x							
Baseline bio-efficacy and chemical analysis	x							
ITNs distribution	x							
Conducted after baseline								
Adverse effects survey		x						
Monitoring the attrition			x	x		x		x
Monitoring fabric integrity for damage			x	x		x		x
Monitoring bio-efficacy using WHO bioassays			x	x	x	x	x	x
Chemical content analysis				x		x		x
Monitoring bio-efficacy using the IACT				x		x

The durability monitoring components that will be evaluated are: attrition, bioefficacy, chemical residual content, and damage to fabric. In addition, the Ifakara ambient chamber testing (IACT) will be conducted as an additional bioassay using nets sampled from the field in Tanzania, 20 times laboratory washed and unwashed nets.

### Study ITNs

Four different products of ITNs will be evaluated: Olyset® Plus, Veeralin®, Tsara® Boost and MAGNet® (Table 3). All test items have been prequalified by the WHO [39]. To ensure households and field workers are blinded to intervention allocation during the study, all ITNs will be rectangular in shape, white in colour, and of standard dimensions (190 cm × 180 cm × 150 cm) and will be labelled with a water-resistant six digits numeric self-laminating code attached to one of the six hanging loops. This code will uniquely identify each ITN and contain numbers representing the country, product, and individual net. Each product of ITN will be assessed at baseline in each country to ensure that they meet WHO bio-efficacy criteria before distribution (Table 4) using well-characterized laboratory-reared *Anopheles* mosquitoes [40] in the WHO cone bioassay or WHO tunnel tests if ITN do not meet the WHO cone bioassay criteria [8]. Insecticide content at baseline will be measured using appropriate Collaborative International Pesticides Analytical Council (CIPAC) methods, at an accredited laboratory.

**Table 3:** Characteristics of durability study ITNs

Test item	Material type	Denier	Bursting strength (kPa)	Mesh size	Active ingredient	Estimated AI dose	Treatment method	Manufacturer	WHO status
Tsara® Boost	Polyethylene	130	≥ 400	21 holes/cm^2^	Deltamethrin and PBO	120 mg/m^2^ of deltamethrin and 440 mg/m^2^ of PBO	Incorporated	NRS Moon Netting FZE, United Arab Emirates	[67]
Olyset® Plus	Polyethylene	150	≥ 250	6.45 holes/cm^2^	Permethrin and PBO	800 mg/m^2^ of Permethrin and 400 mg/m^2^ of PBO	Incorporated	Sumitomo Chemical, Japan	[70]
Veeralin®	High-Density Polyethylene	130	≥ 350	13 holes/cm^2^	Alpha-cypermethrin and PBO	216 mg/m^2^ of alpha-cypermethrin and 79.2 mg/m^2^ of PBO	Incorporated	V.K.A Polymers, India	[68]
MAGNet®	High-Density Polyethylene	150	≥ 450	20 holes/cm^2^	Alpha-cypermethrin	261 mg/m^2^ of alpha-cypermethrin	Incorporated	V.K.A Polymers, India	[71]

**Table 4:** ITNs durability components, outcome measured and analysis method

Component	Definition	Test conducted	Outcome indicators	WHO criteria or industry standard	Analysis method^c^
Attrition	Net loss from household through discarding or use for alternative purpose^a^	Households survey	Net presence	–	Logistic regression
Physical integrity	The physical state of the net to estimate bite protection	Count the number, location and size of the hole(s) of max. 3 nets per household	The holed surface area is measured by the proportionate Hole Index (pHI)^b^ [44] Median hole surface area (cm^2^)	pHI 0–64: good pHI 65–642: damaged pHI ≤ 642: serviceable pHI ≥ 643: too torn or unserviceable	Negative binomial regression, depending on distribution
Functional survival [44]	Estimation of nets still in households in serviceable condition	Presence or absence of net	(Number of nets present and serviceable)/(number of nets originally received and not given away or lost to follow up)	Median net survival in years = time point at which the estimate of functional survival crosses 50%	Logistic regression
Biological efficacy	The ability of the net to incapacitate or kill susceptible anopheline mosquitoes after contact with the insecticide	WHO cone/tunnel test using 25 × 25 cm pieces [8]	The proportion of net samples meeting the optimal WHO bio-efficacy criteria	1 h knock-down ≥ 95% or 24 h mortality ≥ 80% or blood feeding inhibition ≥ 90%	Logistic regression
		Ifakara Ambient Chamber Test (IACT) using whole nets	The proportion of mosquitoes dead at 24 h The proportion of mosquitoes not blood-fed	–	Logistic regression
Insecticide content	Amount of active ingredients in the net	Permethrin, Alpha-cypermethrin and piperonyl butoxide: Gas Chromatography with Flame Ionization Detection (GC-FID) Deltamethrin: High-Performance Liquid Chromatography with UV Diode Array Detection (HPLC–DAD)	The proportion of ITNs meeting with WHO specifications at baseline	MAGNet: 6 [4.5–7.5] g/kg alpha-cypermethrin Olyset Plus: 20 [15–25] g/kg permethrin; 10 [7.5–12.5] g/kg PBO Tsara Boost: 3 [2.25–3.75] g/kg deltamethrin; 11 [8.25–13.75] g/kg PBO Veeralin 6 [4.5–7.5] g/kg alpha-cypermethrin; 2.20 [1.65–2.75] g/kg PBO	Descriptive analysis

^a^Nets that are reported as given away, sold or stolen are not in the denominator (lost to follow up)

^b^Proportionate Hole Index (pHI): the number of holes of each size category measured in the field are weighted by the approximate surface area of the holes to provide a single measure of damage per net

^c^Analyses will include product, time point and village. Where more than one net per household is included in the analysis, a random effect for household will be included in the regression model

### Community sensitization

Community sensitization will be conducted to inform community leaders and community members about the study objectives, study rationale and to request their co-operation during study implementation. All attendees of the community sensitization meetings will be encouraged to ask questions from the study investigators to enable the community to understand the study objectives and the risks and benefits of study participation.

### Baseline surveys and distribution of ITNs

Upon obtaining written informed consent from the head of household or adult resident, field workers that have been trained on the study protocol and procedures will conduct the baseline questionnaire as outlined in Lorenz et al. [41] and will distribute the nets required for each household with one net distributed per sleeping space. Non-study nets found in the households will be withdrawn from households to ensure only study ITNs will be present and used in the households. In Tanzania, the withdrawn nets will be stored in bags labelled with the name of the household head and the study village to be returned to their respective households after the study, while the withdrawn nets will be discarded in Côte d’Ivoire and India following specific country procedure. Handling of withdrawn nets is different between countries because of differences in ethical considerations and regulations between countries.

The questionnaire forms will be written in local languages; Kiswahili for Tanzania, French for Côte d’Ivoire and Telugu for India, and will be pre-loaded in Open Data Kit (ODK) Collect software installed on a hand-held Samsung Tab A tablet computer. Data collected during the baseline surveys will be used as the household roster (names, ages, sex, education, occupation and the relationship to the household head), household wealth indicators [42], house characteristics, number of sleeping areas, ownership of mosquito nets, use of mosquito nets, net attitude indicators [43] and household coordinates in the global positioning system (GPS).

## Randomization to study arms

### ITNs distribution

After the baseline survey questionnaire has been completed, on the same day one out of the four products of ITNs will be assigned to the household based on block randomization using a lottery method. All net types are packaged in identical cloth bags. Each field interviewer is given a large bag containing all four products that they select at random and once the net is selected, additional nets of the same product (starting with the same digit code) will be provided to cover all sleeping areas in the household.

### Generating master lists for follow-up: attrition, fabric integrity and bio-efficacy

The unique identifiers (UID) consist of six numbers generated by the study statistician—the first number identifies the country, the second identifies the treatment arm and the remaining four numbers identify nets distributed per arm. At baseline, the UID of each ITN given to the household will be recorded in the baseline questionnaire and in the ITNs master list with each unique net identification code linked to the household identifying codes and GPS coordinates. Two lists will be generated using a random number generator from the household masterlist: (1) attrition and fabric integrity monitoring masterlist (A-list) and (2) destructive sampling for bio-efficacy and chemical retention monitoring masterlist (B-list). These are separate because bio-efficacy and chemical retention monitoring will require ITNs to be removed from the cohort and replaced with new nets. For attrition and fabric integrity monitoring, the same ITNs will be followed up to the end of the study period unless the household is lost to follow-up or withdraws consent.

### Follow-up surveys

Study ITNs per household visited during attrition monitoring at 6, 12, 24 and 36 months will be inspected for fabric integrity. All study ITNs listed in the master list provided will be inspected while non-study nets found in the household will only be recorded. In the households selected for bio-efficacy testing, the first ITN listed will be withdrawn, and if that ITN is not present in the household, the second ITN on the list provided will be sampled. If no study ITNs are present in the household, another household with the same net product will be visited as a replacement.

#### Adverse effects and ITN usage

One month after distribution, 250 households for each ITNs product will be selected randomly from the household master list for the assessment of ITN usage and adverse events. The head of the selected households will be interviewed on perceived adverse effects from all members using the adverse effect questionnaire adapted from the WHO guideline [8].

#### Monitoring attrition

Attrition (the proportion of ITNs no longer found in their respective households) will be assessed at 6, 12, 24, and at 36 months after distribution. After 6 months, 2712 individual households (678 per product per location) will be selected randomly from the A-list prepared for each country separately. The same households will be followed up longitudinally at 12, 24 and 36 months. The presence or absence of all the ITNs that were distributed will be recorded and if absent, the reasons for absence in the household will be provided by the head of the household. ITNs missing due to wear and tear will be considered as lost due to attrition, and those missing because they were stolen, sold or given away to others, will be considered as lost to follow-up.

#### Monitoring fabric integrity

Each ITN found during the attrition monitoring will be draped over a collapsible net frame [41] and the number of holes of four different sizes categories [8] will be counted by their locations on the net, recorded in a hole tally sheet and entered in the follow-up questionnaire. The locations of the net are obtained by dividing the side panels into four equal zones from top to the bottom and a roof as a fifth location of the net [41]. The four-size categories of the holes are 0.5–2 cm, 2–10 cm, 10–25 cm, and above 25 cm for sizes 1, 2, 3 and 4, respectively approximated using a thumb, fist, head and larger than a head [44]. The overall physical condition of the net will be obtained by weighting the number of holes of each size by 1, 23, 196 and 576 for the four-size categories of holes (based on the average assumed surface area for each size category) to obtain a proportionate hole index (pHI) following WHO guidelines [8]. The pHI value obtained will be used to classify the net as serviceable if the pHI value is less or equal to 642 and too torn if the pHI value obtained is greater than 642 [44].

Additionally, three new nets per product from the same production batch used in this study will be assessed for the Resistance to Damage (RD) score [31] at CITEVE (Centro Tecnológico das Indústrias Têxtil e do Vestuário de Portugal). The RD score is a quantitative metric based on four standardized textile tests taking into account different mechanisms of damage to ITNs including both human factors for damaging the net and laboratory testing data [45, 46]. The average value of standardized laboratory textile test data for snag strength, bursting strength, abrasion and hole enlargement [47] is divided by four so that each laboratory parameter contribute equally to the overall RD value which is expressed in percentage with a value ranging between 0 and 100 [31].

### Monitoring bio-efficacy

#### Mosquito species

Pyrethroid susceptible and pyrethroid-resistant strains will be used for bio-efficacy testing in each country. Mosquitoes are maintained following MR4 guidance [48] by feeding larvae on Tetramin fish food and adults on blood meal between 3 and 6 days after emergence and 10% glucose solution ad libitum. All mosquitoes are maintained inside cages in different rooms to prevent cross-contamination. Temperature and humidity within insectaries are between 27 °C ± 5 °C and 70% ± 20% relative humidity, respectively.

In Tanzania, metabolic pyrethroid-resistant *Anopheles arabiensis* Kingani strain and a full pyrethroid susceptible *Anopheles gambiae *sensu stricto (s.s.) Ifakara strain will be used. In Côte d’Ivoire, metabolically resistant *An. gambiae* s.s. Tiassalé strain and a fully pyrethroid susceptible *An. gambiae s.s.* Kisumu strain will be used. In India, a pyrethroid-resistant *Anopheles culicifacies* strain and a fully pyrethroid susceptible *Anopheles stephensi* strain will be used. The bio-efficacy outcomes will be reported separately for each strain in each country. The susceptibility status of the susceptible and resistant strains will be confirmed at least twice per year in each country following standard procedures [49, 50] during the study period. If the susceptibility status changes during the study, a new colony will be established by either selecting resistant mosquitoes from a mixed colony or re-establishing a new colony from the original source.

#### Mosquito net sampling and preparation

Cone bioassays will be conducted at baseline using 30 nets per product from B-list to ensure nets are of sufficient quality before distribution. At baseline, five net pieces (25 cm × 25 cm) will be cut from position 1 to position 5 of the net as per WHO guidelines [8] and all net pieces will be tested in cone bioassay [38]. For each sampled net from the field, four net pieces (25 cm × 25 cm) will be cut from position 2 to position 5 of the net and tested in cone bioassay. A piece from position 1 (the bottom of the net) will not be cut, as it may be exposed to excessive abrasion due to tucking under the bed [38].

Bio-efficacy testing will be conducted on a total of 30 nets (one from each of 30 households) sampled at random from the B-list for each product at 6, 12, 18, 24, and 30 months post-distribution survey. Only at 36 months, 50 nets per product will be sampled for bio-efficacy and chemical testing estimates. However, 60 (30 selected for bio-efficacy testing and a further 30) nets per product will be sampled at 12 and 24 months for additional testing in the IACT [51]. If the listed ITN is not present, another net (of the same product) within the same household will be selected. After a net is withdrawn for bio-efficacy testing, the household will not be eligible for a next round of bio-efficacy sample collection from the B-master list. The withdrawn net samples will be replaced. The head of the household will be offered any of the ITN products in the trial and allowed to select their replacement based on preference, and this choice will be recorded.

#### WHO cone test procedure

The WHO cone bioassays will be conducted separately at the (1) Vector Control Product Testing Unit (VCPTU) in Bagamoyo, Tanzania, (2) Swiss Centre for Scientific Research (CSRS) in Tiassalé district, Côte d’Ivoire, and 3) ICMR-National Institute of Malaria Research (NIMR) field unit in Bengaluru, Karnataka state, India. Four cones will be attached to each net piece and 5 mosquitoes exposed per cone, 100 mosquitoes per strain at baseline and 80 mosquitoes per strain at each post-distribution survey to each ITN [8]. Non-blood-fed, 2–5 days old, female mosquitoes each of the susceptible and resistant strains for the PBO nets and susceptible strain for the pyrethroid only net will be exposed to the ITN for 3 min [8]. After the exposure, mosquitoes will be removed and kept in holding cups provided with 10% glucose or sucrose solution. Mosquito knockdown will be recorded after 60 min (KD_60_) and mortality after 24-h (M_24_). ITNs that do not meet WHO bio-efficacy thresholds of KD_60_ ≥ 95% or M_24_ ≥ 80% with the susceptible mosquito strains will be tested in the WHO tunnel test [8].

#### WHO tunnel test

Each site will perform tunnel tests independently on each ITN that did not meet WHO efficacy criteria (i.e., ≥ 95% KD_60_ or ≥ 80% M_24_) in the cone test against the susceptible strains of mosquitoes, only one out of four net pieces will be selected for the tunnel test. This is the piece that gave mortality closest to the average mortality in the cone test of the four pieces (i.e., average for that net). The selected piece will be fixed in the tunnel for testing. One tunnel with untreated netting will be used as a negative control. Fifty non-blood fed female susceptible *Anopheles* mosquitoes aged 5–8 days sugar starved for 6–8 h will be released in a tunnel (square section 25 cm × 25 cm) made of glass, 60 cm in length [8]. For pyrethroid only nets, susceptible mosquitoes will be used, while for PBO nets, both susceptible and resistant strains will be used.

At one-third of the length, the netting sample is fixed, with the surface of netting available to mosquitoes of 400 cm^2^ (20 cm × 20 cm) with nine holes each 1 cm in diameter: one hole is located at the centre of the square; the other eight are equidistant and located at 5 cm from the border. In the shorter section of the tunnel, a small rabbit, which will be restrained and unable to move but available to mosquitoes, will be placed as bait. To minimize discomfort to the rabbits, all applicable experimental animal welfare procedures will be adhered to following specific country regulations. In the cage at the end of the longer section of the tunnel, 50 female mosquitoes [52] will be introduced at 18:00. The following morning from 09:00, the mosquitoes will be removed using a mouth aspirator and counted separately from each section of the tunnel and mortality and blood-feeding rates will be recorded. During the test, ambient conditions will be maintained at 27 °C ± 5 °C and 60–100% relative humidity. Acceptable feeding success and mortality in controls will be ≥ 50% and ≤ 10%, respectively.

#### Chemical residual analysis

The residual chemical concentration will be estimated for all products at baseline and after every 12 months using nets allocated in B-list. At baseline, five net pieces (25 cm × 25 cm) will be cut from 30 different mosquito nets (position 1 to 5 of each net) per product and at 12, 24 and 36 months, four net pieces will be cut from position 2 to 5 from the nets sampled for bio-efficacy testing of each product [8]. These pieces will be rolled up and placed in a labelled aluminium foil and stored at 4 °C until they are shipped for chemical analysis following CIPAC methods as described in the WHO specifications for each ITN to check if the chemical concentrations are within the tolerance limit as per the manufacturer’s specifications [Tsara Boost: 333/LN/(M)/3, 33/LN/(M)/3 [53]; Veeralin: 454/LN/(M)/3, 33/LN/(M)/3 [54]; Olyset Plus 331/LN/(M)/3, 33/LN/(M)/3 [55]; MAGNet 454/LN/(M)/3] and within range after operational use [56].

#### Mortality and blood-feeding for PBO ITNs in Ifakara Ambient Chamber Test

Whole ITNs returned from the field at 12 and 24 months for bio-efficacy testing in Tanzania and unwashed and 20 times laboratory washed nets of each product will be evaluated in 18 chambers of the Ifakara Ambient Chamber Test (IACT) in Tanzania [41, 57]. Overall, the 18-arm study will include 4 arms of field-aged nets, 4 arms of unwashed and 4 arms of twenty-times laboratory-washed nets of the brands: Olyset® Plus, Veeralin®, Tsara® Boost and MAGNet® and two untreated nets (negative controls) and two additional positive control nets: PermaNet® 3.0 and standard Olyset® nets both unwashed and twenty-times washed. Unwashed, twenty-times washed nets, positive and negative control nets will be deliberately holed with six holes (4 × 4 cm) as per guidance [8]. Eighteen volunteers will rotate sequentially among the 18 chambers of the IACT nightly based on a prepared roster to allow equal sleeping under nets per product among volunteers. Each chamber of the IACT will have a bed net frame over which the ITN will be draped and a foam mattress upon which one volunteer will sleep. The study will run for seventy-two experimental nights per time point (12 months and 24 months). After 54 experimental nights, each unwashed, 20 times washed nets and positive controls will be replaced with field aged nets for the last 18 nights from the IACT to allow four field aged nets of each product tested for each of the remaining 18 experimental nights, leading to 126 replicates per field-aged product in total. This is done to allow a sufficient sample size for the non-inferiority comparison of field aged nets against Olyset® Plus field-aged net.

IACT allows the testing of multiple strains of mosquitoes at the same time. To understand the bio-efficacy of ITNs on the susceptible and resistant mosquitoes, fifteen nulliparous, sugar starved for 6–8 h, female susceptible *An. gambiae* s.s. Ifakara strain and a resistant *An. arabiensis* Kingani strain mosquitoes will be released at 21.00 h in each of the compartments occupied by one volunteer sleeping under one of the tests ITNs or a negative control net. These species are morphologically identical; thus, one strain will be marked with a fluorescent dye that does not affect their survival or behaviour [58]. At 06.00 h, each volunteer will collect mosquitoes inside the compartment using a mouth aspirator in paper cups. Recaptured mosquitoes will be sorted by species and recorded as fed alive, fed dead, unfed alive and unfed dead, then will be provided with 10% sucrose solution, and held under standard laboratory conditions to assess delayed mortality at 24-h.

### Sample size

#### The sample size for the ITN surveys

The sample size was calculated based on the primary outcome measure of net attrition together with additional nets for the bio-efficacy components. Assuming an average of 2.7 nets per household and a coefficient of variation of 0.25, the formula on page 110 of Hayes and Moulton [59] gives a sample size of 678 households per arm to detect the difference in attrition between two products assuming 3-year attrition rates of 47.5% and 52.5% with 80% power. An additional 260 households for bio-efficacy, chemical analysis and IACT have been added to 678 making a total number of 938 households. Since the median lifespan of ITNs products from durability studies in Tanzania is less than 3 years [29, 60, 61], a 41% loss to follow-up of households is assumed making addition of 657 households to the 938 households. The final total number of households that will be recruited at baseline per product per country is 1595. With an average of 2.7 nets per household [62], the total number of ITNs required per product per country will be 4521 including 5% for error due to poor quality or nets with open seam prior to distribution, Table 5.

**Table 5:** Description of the numbers of ITNs distributed in the study area

Households for longitudinal attrition and fabric integrity monitoring	678
Households for I-ACT, cone bioassay and chemical testing	260
Unadjusted total households per product per country	938
Loss to follow up households	657
Adjusted total households per product per country	1595
The average number of ITNs per household per product	2.7
Total ITNs per product	4305
5% error and nets for replacement	215
Total ITNS required for each product per country	4521

The same net used in the IACT will be used for cone bioassay and chemical testing

#### The sample size for the entomological outcomes in the IACT

Simulation was used to determine the sample size for the bio-efficacy testing of ITNs in the IACT. Each PBO net product will be assessed for non-inferiority against Olyset® Plus on mortality at 24 h. Each product will have sixty nets some tested more than one times for 72 nights with 15 mosquitoes per strain per chamber per night. From a previous study (author, pers.commun.), the variation between individual field-aged nets per product was assumed at a standard deviation of 0.1 and between chamber-nights at 0.15, both on the log-odds scale. Assuming mortality of 70% for the PBO nets and Olyset Plus, 1000 simulation trials were conducted to assess non-inferiority for each trial and the power estimated by the proportion of trials that showed non-inferiority where the product was indeed non-inferior, was over 80%.

To assess the equivalence of field nets to 20-times washed nets on mortality, each product consisting of sixty replicates of field-aged nets as well as four replicates of twenty times laboratory washed and unwashed nets will be tested 54 times (three rounds with 18 replicates each). Assuming 70% mortality for each product of field and washed nets, and assuming 0.1 standard deviation between individual replicate nets per product and a chamber-night standard deviation of 0.15 on the log-odds scale, using 15 mosquitoes per strain per chamber per night, the study is powered at 80%.

#### Samples size for chemical analysis

The baseline quality assurance of the ITNs used in the durability trial is conducted by chemical analysis and biological efficacy testing. A problem with the batch is detected if one or more of the sampled nets fails to meet the acceptance criteria. Since the same batch of nets are tested in three study countries, 10 ITNs are evaluated per site to give a total sample size of 30 ITNs tested at baseline as per WHO guidelines [8]. During each of the subsequent survey time point, 30 net samples will be sampled for chemical analysis as per WHO guidelines [8].

### Data management

Field data will be collected using electronic data capture format in Open Data Kit (ODK) Collect. The data will be sent to the secure server located at Ifakara Health Institute in Tanzania. Cleaned data sets will be returned to CSRS and NIMR India. The information from the baseline questionnaire will be linked to the follow-up surveys using the unique household and country identification number as well as unique net identification numbers. Data collected on paper forms in the laboratory and in the IACT will be entered in excel using double-entry to facilitate cross-referencing and validation. The cleaned excel file of the data will be uploaded to the IHI server. Access to the data on the server will be limited to the data manager.

### Data analysis

All data analyses will be conducted using STATA software (Stata Corp LLC, College Station TX, USA). The durability outcomes; attrition, fabric integrity, bio-efficacy and insecticide content will be estimated (Table 4). These outcomes will be estimated for each net product by survey period and country. A pooled estimate from three countries will also be presented, if appropriate.

The non-inferiority of Veeralin® and Tsara® Boost nets to Olyset® Plus will be carried out using logistic regression for the binary outcome of mosquito mortality with ITN product, compartment, volunteer and day as covariates. A random effect for chamber-night will be included. The 95% confidence interval for the estimated odds ratio for the effect of Tsara Boost® and Veeralin® compared to Olyset® Plus will be presented. Non-inferiority is shown if the confidence interval excludes an unacceptably worse performance. The bound of the margin of non-inferiority for mortality will be set at 0.7. If the lower bound of the confidence interval for the effect of the candidate net compared to Olyset® Plus is greater than 0.7, then we will conclude that the net product is non-inferior [36].

The utility of washed nets as a proxy for field nets will be assessed on the mortality and blood-feeding inhibition endpoints using logistic regression. The estimated odds ratios for the effect of washed nets compared to the negative control, and field-aged nets compared to the negative control will be presented. The ratio of these odds ratios will be estimated along with the 95% confidence interval using interaction terms in the logistic regression model.

## Discussion

This study is the first large-scale prospective household randomized controlled trial of PBO ITNs in three different countries simultaneously. It will allow a pooled estimate of ITN durability from three countries, enabling precise estimates of product performance over time.

In a cluster randomized trial that assessed the effectiveness of Olyset® plus, there was a high washout of PBO concentration within 21 months of use under operational conditions [19] and more than 97% washout after 3 years [63]. Similarly, Olyset® Plus lost more than 87% of PBO concentration after 3 years in Kenya [64]. In Uganda, more than 55% of PBO concentration in Olyset® Plus was washed out within 25 month [65]. These publications highlight the importance of describing the estimated period of performance of PBO ITNs in different contexts. This study will report the persistence of PBO and pyrethroid concentrations in Olyset® Plus, Veeralin®, and Tsara Boost® beside pyrethroid concentration in MAGNet® for the period of 3 years of use in the field in three locations. It will also monitor the added value of PBO against resistant strains of mosquitoes over time as recommended for durability monitoring of products designed to kill pyrethroid-resistant malaria vectors [66].

PBO ITNs including Veeralin® and Tsara® Boost are currently recommended as pyrethroid only ITNs [67, 68] and are required to demonstrate non-inferiority using 24-h mortality and feeding inhibition endpoints compared to the first-in-class Olyset® Plus [69]. This study will measure the non-inferiority of Veeralin® and Tsara® Boost compared to the Olyset® Plus, collected from the field operational settings i.e. the non-inferiority as part of product durability evaluation. Currently, twenty times washed, deliberately holed ITN is used as a reasonable proxy for a field aged net. This study will explore whether this assumption is acceptable, or if it should be revised.

The results of this trial will provide robust and rigorous evidence of the likely replenishment interval, quality and additional bio-efficacy of PBO ITNs compared to pyrethroid only ITNs up to 3 years after the distribution.

## Current study status

This study has finished baseline household data collection and the distribution of ITNs in the study areas in all countries. Currently, follow-up households' data collection and ITNs durability monitoring is ongoing in all study countries. The last follow-up household data collection and ITNs monitoring is expected to end in October 2023.

## Data Availability

Not applicable.

## Abbreviations

CIPAC: Collaborative International Pesticides Analytical Council
GPS: Global positioning system
IACT: Ifakara Ambient Chamber Test
ICF: Informed consent form
IRB: Institutional Review Board
ITNs: Insecticide-treated nets
KD_60_: Knockdown after 60 min
LLINs: Long-lasting insecticidal nets
M_24_: Mortality after 24 h
ODK: Open Data Kit
PBO: Piperonyl-butoxide
pHI: Proportionate hole index
UID: Unique identifiers
WHO: World Health Organization
